# Verification of sleep scales as predictors of suicidal ideation in Japanese dayworkers: a longitudinal study

**DOI:** 10.1007/s41105-022-00404-6

**Published:** 2022-07-22

**Authors:** Yuuki Matsumoto, Naohisa Uchimura, Tatsuya Ishitake, Osamu Itani, Yuichiro Otsuka

**Affiliations:** 1 Division of Public Health, Department of Social Medicine, Nihon University School of Medicine, 30-1 Oyaguchi, Kami-cho, Itabashi-ku, Tokyo, Japan; 2 Department of Neuropsychiatry, Kurume University School of Medicine, Kurume, Japan; 3 Department of Environmental Medicine, Kurume University School of Medicine, Kurume, Japan

**Keywords:** Sleep, Suicidal ideation, Occupational health, Longitudinal studies

## Abstract

The objective of this study was to verify whether the assessment of poor sleep based on two sleep scales could predict suicidal ideation in Japanese dayworkers. A longitudinal survey was conducted among 446 Japanese dayworkers working at a company in Japan. Questionnaires were administered in 2013 (baseline) and 2014 (follow-up). To evaluate suicidal ideation, we used Question 19 of the Self-rating Depression Scale, categorizing participants who chose response options “some/a good part/most of the time” as suicidal ideation (+), and those who chose option “never or only a little of the time” as suicidal ideation (−). Two sleep scales, the Pittsburgh Sleep Quality Index (PSQI) and the 3-Dimensional Sleep Scale (3DSS), were selected as potential predictors of suicidal ideation. Only participants who were suicidal ideation (−) at baseline were included in the analysis, and suicidal ideation (+) individuals at follow-up were considered to have developed suicidal ideation during the interval between baseline and follow-up surveys. Data from 293 participants (236 men, 57 women) were analyzed. Twenty-two participants (7.5%) became suicidal ideation (+) at follow-up. Multiple logistic regression analysis showed that only sleep quality of the 3DSS subscale significantly predicted suicidal ideation, even after adjusting for depressive symptoms. That is, assessment of poor sleep did not predict suicidal ideation when based on the PSQI, but did when based on the 3DSS. Assessment of sleep quality based on the 3DSS may prove more useful in predicting worker suicidal ideation in industrial settings with limited facilities than PSQI.

**Supplementary Information:**

The online version contains supplementary material available at 10.1007/s41105-022-00404-6.

## Introduction

In 1998, the collapse of several major financial institutions contributed to a rapid increase in the suicide rate in Japan [1]. This rate subsequently remained relatively high, making Japan infamous for its high suicide rate. The Basic Act on Suicide Prevention was enacted in 2006, and gradually decreased the suicide rate. However, while the suicide rate in the elderly has decreased markedly, that of the productive population has improved only slightly [1]. Measures to prevent suicide in Japanese workers are thus urgently desired.

Suicide involves a process, with suicidal ideation as a step before the execution of a suicide attempt [2]. Many factors have been shown to be associated with a risk of suicidal ideation, including poor sleep. Sleep is also a common problem for workers. Identification of sleep problems is thus considered useful as a screening index for suicide risk, because sleep problems are easier to answer honestly than mental health symptoms [3, 4]. However, no longitudinal studies on sleep and suicidal ideation appear to have been conducted on Japanese workers.

Many previous studies have demonstrated associations between sleep and suicidal ideation or suicide [4–10], but most have involved cross-sectional research. In addition, many studies have been aimed at patients, adolescents, the elderly and Westerner populations, representing a limitation in applications to Japanese workers. Anna Karin et al. conducted a large, longitudinal study of the general population followed up for an average of 19.2 years [9], but shorter follow-up periods have been described as more appropriate, because proximal risk factors, such as sleep are temporary and variable with aging [11, 12]. Although a longitudinal study of an Asian population was reported by Gunnell et al., that study used a sleep scale with no established reliability or validity [13]. In a longitudinal study, Riveiro et al. demonstrated insomnia as a cause of suicidal ideation among young military personnel [14]. Since sleep items from the Beck Depression Inventory—Second Edition (BDI-2) were used, that study did not conduct a general assessment of sleep, including disturbed rhythms, insomnia, or lack of sleep. Bernert et al. found that suicidal ideation was predicted by decreased sleep efficiency and changes in sleep/wake patterns in studies using polysomnography and actigraphs [15, 16]. However, those kinds of sleep investigation are difficult to apply in a population approach targeting workers. Therefore, when trying to prevent suicides by screening for sleep problems among populations in office settings, simpler methods that can assess and manage sleep without using specialized medical equipment are needed.

Sleep scales such as the Pittsburgh Sleep Quality Index (PSQI) and the Athens Insomnia Scale (AIS) are typical examples of easy sleep evaluations. The PSQI, which is the most popular sleep scale in sleep research, can measure and score insomnia, including nighttime and daytime symptoms [17, 18]. However, it displays some problems. For example, daytime symptoms can occur not only with insomnia, but also with insufficient duration of sleep [19]. In addition, whereas a 24-h society has developed, mental health disorders can reportedly be caused by the resulting fluctuations in sleep/wake patterns (social jet lag) on weekdays and weekends [20, 21]. Because people in Japan already have shorter sleep duration than people in other countries [22], they may be more likely to develop problems associated with social jet lag and insufficient sleep. Therefore, the PSQI may thus be inadequate as a method of evaluating modern Japanese sleep.

We have previously described the development of a new scale, the 3-Dimensional Sleep Scale (3DSS). This scale can score and evaluated nighttime and daytime symptoms of sleep problems separately, in addition to fluctuations in sleep/wake patterns [23, 24]. Furthermore, in a cross-sectional study, we demonstrated that individuals judged as having poor sleep by the 3DSS showed an odds ratio for suicidal ideation about 3–6-times higher than those judged as having good sleep [25]. Nevertheless, since that study was not longitudinal, a temporal causal relationship could not be established. We, therefore, conducted this longitudinal study of Japanese workers to verify the following two hypotheses: that sleep assessment by the 3DSS can predict suicidal ideation; and it is superior to PSQI as a predictor of suicidal ideation.

## Methods

### Participants

Baseline data were collected in June 2013 and follow-up data were collected in June 2014, with the cooperation of a workplace in Japan in which one of the coauthors was engaged in occupational health activities. The workplace was mainly manufacturing establishments, where approximately 70% of the employees were blue-collar and 30% were white-collar. The present study was conducted anonymously after creating an ID for the longitudinal study. Paper questionnaires were distributed by the occupational health or personnel department staff in the workplace. Questionnaires were answered anonymously in the workplace, with the supervisor asked to collect the completed questionnaires within a few weeks using a collection box, then to return them to us via mail. Eligible participants comprised 446 Japanese employees, of whom 443 returned the questionnaire (response rate: 99.3%).

### Measures

Regarding demographic characteristics, we collected data from participants on age, gender, and marital status (married, unmarried, others). Since this study used a sleep scale designed for dayworkers, we also asked about work styles as a step to exclude shift workers.

The self-rating depression scale (SDS) was used as an indicator of depressive symptoms [26–28]. The SDS consists of 20 questions, including appetite, sexual desire, sleep, physical complaints, hopefulness, and suicidal ideation. Questions are answered by selecting one of four response options: (1) never or only a little of the time; (2) some of the time; (3) a good part of the time; or (4) most of the time. The higher the score, the more serious the depression. Littlewood et al. used the total score of the depression scale after excluding the scores for sleep and suicidal ideation as confounding factors when examining the relationship between sleep and suicidal ideation [29]. Referring to that study method, we used the total score for 18 items of the SDS after excluding the two items related to sleep (Question 4) and suicidal ideation (Question 19) as adjustment variables for analysis.

We used the two sleep scales, the PSQI and 3DSS, to measure outcome variables. The 3DSS was designed for use by Japanese dayworkers and its reliability and validity have been established [23]. The 3DSS comprises three categories (sleep phase, sleep quality, and sleep quantity), each with 5 items (for a total of 15 items). All items ask about usual sleep habits during the preceding month. The sleep phase items are as follows: (1) “I go to bed at a fixed, regular time on weekdays and weekends;” (2) “I wake up at a fixed, regular time on weekdays and weekends;” (3) “I have a well-balanced breakfast every day;” (4) “‘Morningness’ is better suited to me than is ‘eveningness’;” and (5) “What time do you wake up on weekdays?” The items assessing sleep quality are as follows: (6) “It takes me more than 30 min to fall asleep;” (7) “I wake up more than twice a night;” (8) “I wake up earlier than usual (over 2 h) and cannot fall asleep again;” (9) “I don’t sleep soundly;” and (10) “I worry that I cannot fall asleep.” Finally, the items for sleep quantity are as follows: (11) “I sleep for less than 6 h on weekdays;” (12) “I cannot get enough sleep even though I want to;” (13) “I don’t feel free from sleepiness or fatigue when I wake;” (14) “I feel sleepy not only in the afternoon, but also in the morning and/or evening;” and (15) “I often doze off.” To answer these items, the respondent selects one of the four responses to best fit their sleep habits, as follows (except for Item 5): (1) always, (2) often, (3) rarely, and (4) never. For Item 5, the options are: (1) about 6:00 a.m. or earlier; (2) about 6:30 a.m.; (3) about 7:00 a.m.; and (4) later than 7:00 a.m. For scoring, answers of 1–4 are given scores of 3–0, respectively, for the sleep phase items (Items 1–5), and scores of 0–3, respectively, for the sleep quality and sleep quantity items (Items 6–15). The score for each category ranges from 0 to 15, with higher scores indicating a better result for that sleep condition. The cutoff values reported in previous studies were used in this study [24]. The cutoff values for sleep phase, sleep quality, and sleep quantity are 8/9, 10/11, and 8/9, respectively; < 9/ < 11/ < 9 to indicate poor sleep phase/quality/quantity, respectively, and ≥ 9/ ≥ 11/ ≥ 9 to indicate good sleep phase/quality/quantity, respectively. “Poor sleep phase” means irregular and delayed sleep rhythm, “poor sleep quality” means low sleep efficiency with symptoms of nocturnal insomnia, and “poor sleep quantity” indicates the presence of daytime arousal disorders associated with insufficient duration of sleep.

The PSQI was developed by Buysse et al. in 1989, as a measure of overall sleep (Subjective sleep quality, Sleep latency, Sleep duration, Habitual sleep efficiency, Sleep disturbances, Use of sleeping medication, and daytime dysfunction) and has been used in many previous studies [17]. The Japanese version was developed by Doi et al. and its reliability and validity has been confirmed [18]. The cutoff score was set at 5/6, with this study defining good status for scores < 6 and poor status for scores ≥ 6.

### Definitions

To evaluate suicidal ideation, we used Question 19 of the SDS: “I feel that others would be better off if I were dead.” Mizuno demonstrated a risk of suicide among Japanese individuals who selected a response choice of (2), (3), or (4) for this question [30]. We, therefore, identified participants who chose responses (2)–(4) for Question 19 as suicidal ideation (+), and those who chose (1) as suicidal ideation (−).

### Statistical analysis

Individuals who were suicidal ideation (−) at baseline but suicidal ideation (+) at follow-up were considered to have developed suicidal ideation during the interval between baseline and follow-up. Only participants who were suicidal ideation (−) at baseline were included in analysis. In univariate analysis, the Chi-square test or Fisher's exact test was performed for nominal variables, while the Mann–Whitney *U* test was performed for continuous variables. In multivariate analysis, multiple logistic regression analysis was performed. To avoid multicollinearity, each sleep scale was entered into a separate regression model as a predictor variable. SPSS version 22 software (IBM Corp, Armonk, NY, USA) was used for analysis, and the level of significance was set at *p* < 0.05. Multivariate analysis was performed using the direct method with 95% confidence intervals (CIs).

### Ethical considerations

We ensured that none of the respondents felt pressured into participating, or suffered consequences due to non-participation, and indicated to them that their response constituted their informed consent to participate. Respondents also received no reward for participation. Personal data were strictly monitored to maintain confidentiality and protect participant privacy. Data were kept and only used for scientific purposes. This study was approved by the ethics review board at Kurume University (approval No. 12065).

## Results

Since the 3DSS is a scale for dayworkers, we excluded 40 shift workers among the 443 participants from whom questionnaires were collected. Another 36 participants with missing data at baseline were also excluded. Moreover, 74 participants categorized as suicidal ideation (+) at baseline were excluded. Finally, total of 293 participants (236 men, 57 women) were included for analysis and none of them dropped out or missing data of suicidal ideation at follow-up. Thus, the valid response rate was 65.7%.

Table 1 shows the characteristics at baseline classified by the presence of suicidal ideation after 1 year. In the follow-up year, suicidal ideation was observed in 22 participants (7.5%). The Chi-square test or Fisher’s exact test showed significant differences in gender and sleep quality from the 3DSS between suicidal ideation (+) and (−) participants. Frequencies of male gender and poor sleep quality were higher with suicidal ideation (+). Otherwise, no significant differences in age, marital status, PSQI score, sleep phase, or sleep quantity were seen. The Mann–Whitney test showed a significant difference in SDS score between suicidal ideation (−) (mean rank, 143.5) and (+) (mean rank, 189.6), and no significant difference in PSQI score and 3DSS scores. In addition, the means and standard deviations for all items of PSQI and 3DSS are shown in the Supplementary material for reference (Online Resource 1).

**Table 1:** Characteristic data at baseline according to presence of suicidal ideation at follow-up

(Baseline)	Total	Suicidal ideation at follow-up; *n* (%)		*p* value
		(−)	(+)	
All				
*N*	293	271 (92.5)	22 (7.5)	
Age	42.0 ± 9.2	42.2 ± 9.3	39.9 ± 8.6	0.164
Gender				
Men	236	215 (91.1)	21 (8.9)	0.048*
Women	57	56 (98.2)	1 (1.8)	
Marital status				
Married	212	199 (93.9)	13 (6.1)	0.342
Unmarried	65	58 (89.2)	7 (4.9)	
Other	16	14 (87.5)	2 (12.5)	
SDS^†^				
Scores	37.7 ± 5.9	37.4 ± 5.9	40.5 ± 5.3	0.014**
PSQI				
Scores	4.7 ± 2.0	4.7 ± 2.0	5.4 ± 2.0	0.120
Good	203	190 (93.6)	13 (6.4)	0.281
Poor	90	81 (90.0)	9 (10.0)	
3DSS				
Sleep phase				
Scores	9.4 ± 2.9	9.4 ± 3.0	9.6 ± 2.8	0.684
Good	188	173 (92.0)	15 (8.0)	0.683
Poor	105	98 (93.3)	7 (6.7)	
Sleep quality				
Scores	10.8 ± 2.7	10.8 ± 2.8	9.9 ± 2.6	0.102
Good	172	165 (95.9)	7 (4.1)	0.008*
Poor	121	106 (87.6)	15 (12.4)	
Sleep quantity				
Scores	8.6 ± 2.8	8.6 ± 2.8	8.3 ± 2.4	0.514
Good	157	146 (93.0)	11 (7.0)	0.726
Poor	136	125 (91.9)	11 (8.1)	

*SDS* Self-rating Depression Scale, *PSQI* Pittsburgh Sleep Quality Index, *3DSS* 3-Dimensional Sleep Scale

Age and Scores show mean ± standard deviation

*Significant difference based on Chi-squared test or Fisher’s exact test

**Significant difference based on Mann–Whitney *U* test

^†^Total score for 18 items of the SDS, excluding items related to sleep and suicidal ideation (Question 4 and 19)

Table 2 shows the multivariate analysis for suicidal ideation including PSQI and 3DSS subscales. No significant increase in odds ratio (OR) for suicidal ideation (+) at follow-up was found for PSQI score, and this result was unchanged by adding the SDS score to the model. In 3DSS subscales, on the other hands, significant increases in the OR for suicidal ideation (+) at follow-up were observed with poor sleep quality in both the model without SDS score (OR: 4.34; 95% CI: 1.55–12.1) and the model with SDS score (OR: 3.34; 95% CI: 1.14–9.79).

**Table 2:** Multivariate regression analysis including the PSQI or the 3DSS subscales for suicidal ideation (+) at follow-up

	Model 1		Model 2		Model 3	
	OR	95% CI	OR	95% CI	OR	95% CI
PSQI						
Good	1.00		1.00		1.00	
Poor	1.62	0.67–3.95	1.66	0.67–4.11	1.15	0.44–3.02
3DSS						
Sleep phase						
Good	1.00		1.00		1.00	
Poor	0.82	0.33–2.09	0.57	0.20–1.60	0.50	0.18–1.44
Sleep quality						
Good	1.00		1.00		1.00	
Poor	3.34*	1.32–8.45	4.34*	1.55–12.1	3.34*	1.14–9.79
Sleep quantity						
Good	1.00		1.00		1.00	
Poor	1.17	0.49–2.79	0.76	0.29–1.97	0.72	0.27–1.89

*PSQI* Pittsburgh Sleep Quality Index, *3DSS* 3-Dimensional Sleep Scale, *SDS* Self-rating Depression Scale

Model 1: Crude, Model 2: adjusted for age, gender, and marital status, Model 3: adjusted for age, gender, marital status, and total score for 18 items of the SDS, excluding items related to sleep and suicidal ideation (Question 4 and 19)

*Significant difference (*p* < 0.05)

## Discussion

This longitudinal study was conducted to clarify whether a subjective sleep scale could predict suicidal ideation among Japanese dayworkers. Most previous studies on sleep and suicidal ideation have used cross-sectional designs to examine Western populations or patients, and no longitudinal studies have been reported for dayworkers in Japan, as a country with a high suicide rate. In addition, two different sleep scales were used to verify the prediction of suicidal ideation. While the PSQI was unable to predict suicidal ideation, the 3DSS subscale; the sleep quality predicted suicidal ideation. Furthermore, industrial staff must work on suicide countermeasures under insufficient environmental conditions, unlike clinical staff. If the occurrence of suicidal ideation can be predicted using a simple method, such as a sleep scale, suicide prevention measures may be much more effectively targeted toward at-risk workers.

In this study, 74 of 367 patients (20.1%) showed suicidal ideation at baseline. In a large-scale survey of 40,436 Japanese people conducted by the Nippon Foundation in 2016, the rate of suicidal ideation was reported as 25.4% [31]. Although direct comparisons are not feasible, because the indicators for measuring suicidal ideation differed between studies, the sample population in this study may not have differed markedly from the national average, or may have been slightly more biased toward healthy individuals. This was because the present study targeted workers in the workplace. In other words, since all participants were healthy enough to attend work, the sample would not have contained the same levels of unemployed or sick individuals present in the general population. Although history of psychiatric disorders was not clarified in this study, the possibility of accidental selection bias due to the inclusion of large numbers of psychiatric patients was considered low.

No previous studies have investigated the PSQI as a predictor of suicidal ideation in workers. The PSQI includes nightmare and sleep efficiency items, and both sleep-related factors have been shown to be predictors of suicidal ideation in previous studies [16, 32–34]. Nevertheless, PSQI score was not a significant predictor of suicidal ideation in this study. The PSQI is a comprehensive sleep evaluation tool that includes various other items, which may have prevented the PSQI score from predicting suicidal ideation.

Of the 3DSS sleep phase scores, sleep quality scores, and sleep quantity scores, sleep quality scores were shown to represent predictors of suicidal ideation. Because all subscales of 3DSS and SDS scores were included in the analysis, sleep quality was a predictor of suicidal ideation even after adjusting for sleep phase (irregular and delayed), sleep quality (lack of sleep duration), and SDS score. Sleep quality score in the 3DSS assesses nocturnal symptoms of insomnia, such as difficulty falling asleep, disturbance of sleep, and earlier morning awakening. In a study of a general population of 5123 American individuals, Tubbs et al. demonstrated a significant association between suicidal ideation and nocturnal symptoms of insomnia, but no significant associations with daytime symptoms of insomnia [10]. Anna Karin et al. demonstrated that insomnia, including several nocturnal symptoms and excluding a daytime symptom, was significantly predictive of suicide in a large longitudinal study [9]. Furthermore, the strength of the association was reduced by adjusting for depressive symptoms, but remained significant. These two previous reports are consistent with the present results, and nocturnal symptoms of insomnia are considered more important to predict suicidal ideation than daytime symptoms among Japanese dayworkers.

We also considered the causes of the differing results for the PSQI and 3DSS. The PSQI evaluates a total score for nocturnal and daytime symptoms, while the 3DSS evaluates these separately. Japanese individuals tend to show a shorter duration of sleep, and daytime symptoms such as daytime sleepiness also occur due to a lower sleep duration [19, 22, 35]. Such individuals would show higher PSQI scores despite lacking nocturnal symptoms of insomnia. In particular, when targeting workers who often sleep shorter durations, many individuals with high PSQI scores due to a lack of sleep may be included. This was considered the prime cause of the different results for the PSQI and 3DSS. That is, the 3DSS evaluated differences in sleep more strictly than the PSQI, and thus could have found sleep-related factors representing predictors of suicidal ideation.

Some limitations to this study should be considered. First, the possibility of selection bias is unavoidable, due to the insufficient sample size. The skewed gender ratio, in particular, may be caused on the characteristics of the workplace. Second, we could not sufficiently adjust for confounding factors (e.g., lifestyle factors, such as smoking and drinking, financial background, and medical history), because the sample size was not large enough to adjust for the many potential confounding factors. Third, due to ethical issues, we used the suicidal ideation item of the SDS instead of a suicide-specific scale. Future research should examine larger cohorts to allow adjustment for many confounding factors, and should use a suicide-specific scale.

### Supplementary Information

Below is the link to the electronic supplementary material.

Supplementary file1 (DOCX 20 KB)
