# Supplementary material for: Verification of sleep scales as predictors of suicidal ideation in Japanese dayworkers: a longitudinal study
Source: Sleep Biol Rhythms. 2022 Jul 22;20(4):577–83. doi: 10.1007/s41105-022-00404-6 (PMC10899985; doi:10.1007/s41105-022-00404-6)
Supplement: Supplementary file 1 — Supplementary file1 (DOCX 20 KB) [file 41105_2022_404_MOESM1_ESM.docx]

**Supplementary Material** The scores (mean ± standard deviation) for items C1 to C7 of the PSQI, and 1) to 15) of the 3DSS at baseline

| **(Baseline)** | | **Total** | **Suicidal ideation at follow-up** | |  |
| --- | --- | --- | --- | --- | --- |
|  |  |  | **(-)** | **(+)** | **p value** |
| **PSQI** | C1 | 1.2 ± 0.6 | 1.2 ± 0.6 | 1.3 ± 0.6 | 0.557 |
|  | C2 | 0.6 ± 0.7 | 0.5 ± 0.7 | 0.7 ± 0.7 | 0.186 |
|  | C3 | 1.4 ± 0.7 | 1.4 ± 0.7 | 1.5 ± 0.7 | 0.598 |
|  | C4 | 0.1 ± 0.4 | 0.1 ± 0.4 | 0.1 ± 0.2 | 0.734 |
|  | C5 | 0.6 ± 0.5 | 0.6 ± 0.5 | 0.7 ± 0.5 | 0.627 |
|  | C6 | 0.1 ± 0.3 | 0.0 ± 0.3 | 0.2 ± 0.7 | 0.033* |
|  | C7 | 0.8 ± 0.7 | 0.7 ± 0.7 | 1.0 ± 0.6 | 0.095 |
|  |  |  |  |  |  |
| **3DSS** |  |  |  |  |  |
| **Sleep Phase** | 1) | 2.1 ± 0.9 | 2.1 ± 0.9 | 2.0 ± 0.8 | 0.498 |
|  | 2) | 2.2 ± 0.9 | 2.2 ± 0.9 | 2.2 ± 0.8 | 0.813 |
|  | 3) | 2.0 ± 1.1 | 2.0 ± 1.1 | 2.0 ± 1.2 | 0.782 |
|  | 4) | 2.6 ± 1.0 | 2.6 ± 1.0 | 2.4 ± 0.9 | 0.463 |
|  | 5) | 1.8 ± 0.8 | 1.8 ± 0.8 | 1.9 ± 0.8 | 0.271 |
| **Sleep Quality** | 6) | 3.2 ± 0.8 | 3.2 ± 0.8 | 3.1 ± 0.8 | 0.381 |
|  | 7) | 3.1 ± 0.9 | 3.1 ± 0.9 | 2.8 ± 1.0 | 0.109 |
|  | 8) | 3.5 ± 0.7 | 3.5 ± 0.7 | 3.4 ± 0.7 | 0.412 |
|  | 9) | 2.7 ± 0.8 | 2.7 ± 0.8 | 2.4 ± 0.7 | 0.060 |
|  | 10) | 3.4 ± 0.7 | 3.4 ± 0.7 | 3.3 ± 0.6 | 0.541 |
| **Sleep Quantity** | 11) | 2.5 ± 1.0 | 2.5 ± 1.0 | 2.4 ± 0.8 | 0.807 |
|  | 12) | 2.6 ± 0.8 | 2.6 ± 0.8 | 2.6 ± 0.9 | 0.630 |
|  | 13) | 2.8 ± 0.7 | 2.8 ± 0.8 | 2.6 ± 0.7 | 0.135 |
|  | 14) | 2.7 ± 0.7 | 2.7 ± 0.7 | 2.9 ± 0.6 | 0.288 |
|  | 15) | 3.1 ± 0.8 | 3.1 ± 0.8 | 2.9 ± 0.7 | 0.247 |

* Significant difference based on Mann-Whitney U test.
